# The Longest Known Survival of a Patient With Bioprosthetic Aortic Valve Replacement: A 42-Year Follow-Up

**DOI:** 10.7759/cureus.44069

**Published:** 2023-08-24

**Authors:** Khudheeja A Ahmed, Juwayria Ahmed, Anoushka Samant, Yusra Arub, Ibrahim Mohsin, Mohammed Habeeb Ahmed

**Affiliations:** 1 Department of Research, KAAJ Healthcare, San Jose, USA; 2 Department of Internal Medicine, Norton Community Hospital, Norton, USA; 3 Department of Cardiology, KAAJ Healthcare, San Jose, USA

**Keywords:** patient longevity, case report, bioprosthetic, aortic stenosis, aortic valve replacement

## Abstract

Aortic valve replacement (AVR) is a frequently performed procedure for treating aortic valvulopathy. AVR involves replacing the damaged aortic valve with either a mechanical or a bioprosthetic valve. While many factors are involved when selecting between the two options, age and patient preference are the deciding factors at this point. Mechanical valves demonstrate long-standing durability that overlaps with the accompanied bleeding risk due to lifetime anticoagulant therapy, making them a more favorable choice for younger patients. Bioprosthetic valves are preferred for older patients as they show a reduced risk of thrombogenicity. However, bioprosthetic valves have a higher incidence of structural valve degeneration (SVD) than mechanical valves. Our case report focuses on a 76-year-old patient who had undergone an AVR with a bioprosthetic valve at the age of 33, which has still not demonstrated any valve deterioration. As the longest known case of bioprosthetic durability, this patient provides useful data for designing bioprosthetic valves more resistant to structural degeneration and thereby better suited to younger patients or those at higher risk of bleeding.

## Introduction

Aortic valve replacement (AVR) is one of the most common cardiac procedures performed and is used to treat mainly aortic stenosis but can also be used for aortic regurgitation [1]. AVR is the treatment of choice for both symptomatic patients with severe aortic stenosis and asymptomatic patients with impaired left ventricular ejection fraction [2]. The dysfunctional or damaged aortic valve is replaced with one of two options: a mechanical or bioprosthetic valve. This choice depends on a multitude of factors including patient choice, age, the risk of anticoagulation complications, the presence of a small aortic annulus, and conditions that require anticoagulation therapy [3].

Age is a vital factor to take into consideration when choosing between mechanical and bioprosthetic valves for AVR [1]. Mechanical valves are durable and rarely need to be replaced, while bioprosthetic valves show greater rates of structural valve degeneration (SVD) due to the utilization of biological tissue, increasing the risk of reoperation [3]. As a consequence of a more prominent immune response and increased valve calcification in younger patients, most bioprosthetic valves do not reach their average lifespan of 15 years as they do in older patients, with most deteriorating within a decade, especially for patients under the age of 65 [4]. This signifies that structural valve degeneration occurs faster in younger patients, implying that a longer life expectancy increases the risk of multiple reoperations [3]. As a result, the European Society of Cardiology (ESC)/European Association for Cardio-Thoracic Surgery (EACTS) guidelines recommend mechanical valves for the aortic position in patients younger than 60 years, and the American College of Cardiology (ACC)/American Heart Association (AHA) guidelines recommend the same for patients younger than 50 years of age [5].

However, mechanical valves are thrombogenic and therefore require long-term treatment with anticoagulants, specifically warfarin, to prevent clots, leaving patients at risk of bleeding and enforcing diet and activity restraints [1]. To avoid this, bioprosthetic valves that are not or less thrombogenic are recommended for patients at a higher risk of bleeding, as well as for older patients (>60 years old) for whom multiple reoperations are less likely due to shorter life expectancy [4]. Although both European and American guidelines on valvular heart disease recommend the use of mechanical valves to patients under 50, with the European guidelines even extending this recommendation to patients under 60, all age groups have demonstrated an increase in bioprosthetic valve usage over the past few decades [1]. Recent data on long-lasting bioprosthetic valves free from structural valve degeneration, including that of this case study, might guide methods to reduce the incidence of SVD in bioprosthetic valves [1]. Redesigning bioprosthetic valves targeting a younger population provides patients with an option that does not require long-term anticoagulation therapy nor causes an increased risk of bleeding, both common consequences associated with mechanical valve replacement. The advantages of such an improvement include longer life expectancy and a lower rate of reoperation for younger patients, as well as for patient populations that are at high risk of bleeding [1]. The increased long-term durability of bioprosthetic valves would also enhance the longevity of sutureless AVR (SU-AVR) as the procedure utilizes bioprosthetic valves [2].

Achieving this goal requires up to 20 years’ worth of data on the cases of long-term optimal hemodynamic function with no structural deterioration, which is currently unavailable [1]. Additional long-term follow-up studies are required to identify aspects that can improve the durability of bioprosthetic valves in younger patients. This case study is significant as it focuses on a 76-year-old patient who had an AVR 42 years ago at the age of 33. We find that this is the longest known case in the literature of a bioprosthetic valve where SVD has not been called for reintervention. The recent echocardiogram done on May 1, 2023, showed no significant abnormality of the bioprosthetic valve, in terms of neither stenosis nor regurgitation, which attests to the lack of any significant SVD, if any. This case report provides essential data on the durability of bioprosthetic valves for further research regarding degeneration, as well as potential options for improvement [6].

## Case presentation

**Figure 1:**
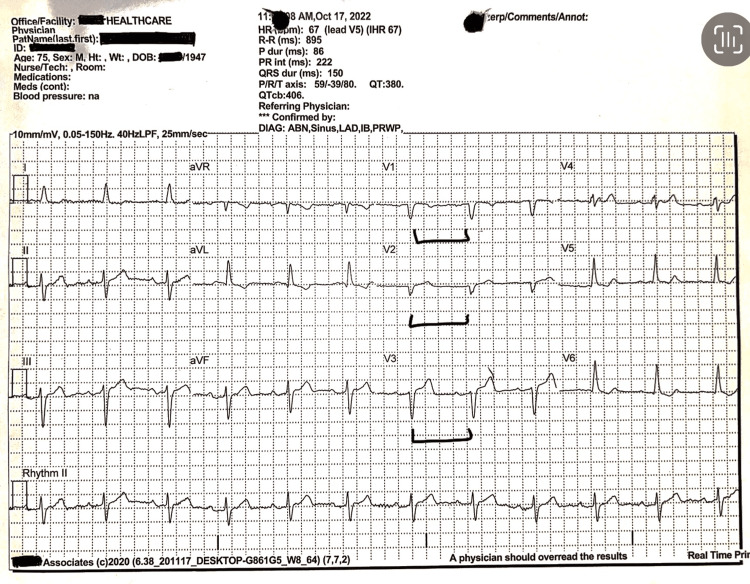
This EKG documents poor R wave progression, as indicated by the brackets. Intraventricular conduction delay is evidenced by the QRS duration (ms) of 150. Left axis deviation is documented in leads 1 and augmented vector foot (aVF).

A 76-year-old male patient presented to the cardiologist’s clinic for a three-month follow-up. He was evaluated by the cardiologist and was deemed clinically stable. The patient has a medical history of hypertension, anemia, arthritis, hyperlipidemia, gout, and valvular heart disease. His current medications include alendronate 70 mg weekly, calcium citrate 1200 mg daily, folic acid 1 mg daily, lisinopril 10 mg daily, atorvastatin 20 mg daily, tamsulosin 0.4 mg daily, vitamin D 50000 IU weekly, and niacin extended release (ER) 1000 mg daily. He is also a former smoker, having quit smoking at the age of 48. As shown in Figure 1, the patient had undergone an AVR in November 1980 with a bioprosthetic valve (St. Jude Medical {SJM} valve prosthesis, model number 27A-101) that still has not experienced any known structural valve degeneration despite being 42 years post-surgery.

**Figure 2:**
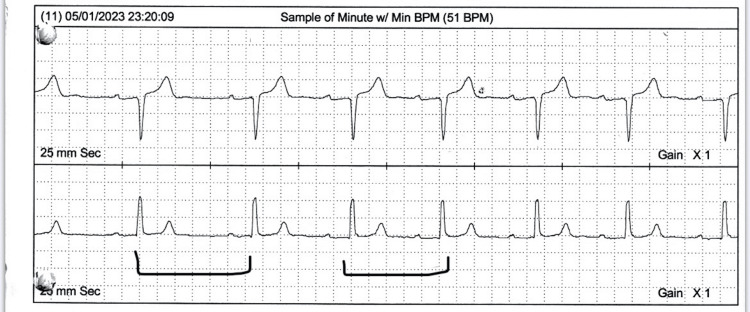
The Holter report indicates marked sinus arrhythmia.

The results from a transthoracic echocardiogram done on May 1, 2023, showed moderate enlargement of the left ventricle with decreased systolic function, global hypokinesis with an ejection fraction of about 35%-40%, mild to moderate left ventricular hypertrophy (LVH), E/A reversal (diastolic dysfunction), asymmetric septal hypertrophy, mild left atrial enlargement, mild mitral regurgitation (MR), mild tricuspid regurgitation (TR), and the prosthetic aortic valve. The aortic valve appears to be functioning adequately and appropriately. An EKG from October 2022 showed sinus rhythm with left axis deviation, poor R wave progression, and intraventricular conduction delay (Figure 1). Additionally, a 24-hour Holter done on May 1, 2023, showed a sinus rhythm with a heart rate ranging from 51 to 120 beats per minute (bpm) with an average of 68 bpm and marked sinus arrhythmias (Figure 2). A carotid ultrasound done on April 2, 2021, showed 20%-39% stenosis of the internal carotid artery (ICA) with antegrade vertebral flow bilaterally. A transcranial Doppler (TCD) ultrasound done on April 2, 2021, showed small vessel disease. An adenosine cardiolite stress test done on April 22, 2021, showed no evidence of adenosine-induced ischemia and an ejection fraction of 62%. For the past seven years, the patient has been undergoing consistent three-month follow-ups at the clinic and has demonstrated long-term clinical and hemodynamic stability, demonstrating the durability and adequate functioning of his bioprosthetic valve such that he has normal activities of daily living without any restrictions.

## Discussion

Since the 1950s, aortic valve replacements have been performed to treat aortic stenosis [3]. Bioprosthetic valves were introduced in a clinical manner in the 1960s as an alternative to the existing assortment of mechanical ball-caged, monoleaflet, and bileaflet valves [3]. Due to a reduced tendency to clot and the ability to eliminate anticoagulant treatment, porcine and bovine bioprosthetic valves have been implemented in AVR for the past several decades [7].

Sutureless AVR (SU-AVR) is a recent advancement that allows for the removal of the damaged valve while eliminating the need for sutures, as well as minimizing aortic cross-clamp and cardiopulmonary bypass (CPB) time, reducing the potential risk of cardiovascular morbidity and mortality [8]. While there is no statistical significance between the mortality rate of patients with sutureless and stented bioprosthetic valves, during concomitant and high-risk procedures, SU-AVR can prove beneficial to minimize complications and morbidity associated with lengthy surgical duration [8]. It is recommended that sutureless AVR be prioritized for older patients and patients with several medical conditions or with anatomical abnormalities that pose a surgical difficulty, such as porcelain aorta or small aortic annulus [2]. Due to the lack of data confirming long-term durability, it is suggested that standard AVR remains the gold standard for patients younger than 75 until relevant trial data has been analyzed and the procedure is deemed long-lasting [2].

Multiple studies indicate that patients younger than 65 years of age experience increased rates of structural valve degeneration after AVR with a bioprosthetic valve, regardless of whether a stented or stentless valve was used [4,9]. Increased rates of SVD subsequently raise the risk of reoperation, which then makes current bioprosthetic valves a less favorable option for younger patients. For the Toronto stentless porcine valve, one study found significantly lower freedom from SVD at 12 years post-procedure for patients younger than 65 years (52%), as compared to the older population (85%) [9]. For the same valve, another study discovered that after a duration of 12 years post-procedure, the rate of SVD for patients younger than 65 was 48%, while patients older than 65 had a significantly lower rate of 15% [4]. Similar results with stented porcine valves found that after a course of 10 years post-procedure, the rate of SVD was significantly higher in bioprosthetic valves than in mechanical valves but only in patients younger than 65; there was no statistically significant difference in patients over 65 years of age [4]. The aforementioned data compiled from numerous studies involving a variety of bioprosthetic valves exemplifies the statistically significant difference between the risk of SVD in older patients (>65) and younger patients (<65). This elucidation further establishes the unfavorable risk of multiple reoperations in younger patients with bioprosthetic valves.

The incidence of SVD restricts the longevity of bioprosthetic valves. One study found that 21.5% of the patients with subclinical SVD either advanced to clinical SVD or underwent additional aortic valve surgery within a 26-month period. These patients tended to be younger and more prone to hypertension, indicating the influence of age on the rate of SVD [10]. Valve leaflet calcification and the type of valve material are major contributing factors to the development of SVD, specifically in younger patients with higher BMIs or at higher risk of cardiovascular diseases post-AVR.

Transthoracic echocardiography (TTE) is the standard method to assess prosthetic valve function and diagnose SVD. Currently, both the European Society of Cardiology and the American Heart Association/American College of Cardiology guidelines recommend establishing a baseline assessment within 6-12 weeks and long-term monitoring via annual TTE starting five or 10 years post-procedure, respectively. However, since follow-ups by repeated TTE are undertaken only if the patient manifests symptoms suggesting valve dysfunction, early subclinical changes in valve performance may be missed, and Rodriguez-Gabella et al. recommend that at least one TTE should be done between the one- and five-year follow-ups followed by annual examinations thereafter [11]. Additionally, a study by Flameng et al. found that the anti-calcification treatment of bioprosthetic valves prior to the surgery resulted in delayed onset and reduced severity of SVD, as well as reduced rates of valve regurgitation by preventing cusp rupture [12]. Based on the results of these studies, bioprosthetic valves should be carefully monitored by regular echocardiography to catch early signs of structural valve degeneration and should also be treated with anti-calcification treatment during the preparation of the valves to prolong valve durability.

Different bioprosthetic valves have been designed to achieve maximum durability in patients. The implementation of the stentless Freestyle bioprosthetic valve, utilized in aortic root replacements, was regarded as successful as it ensured long-standing clinical stability and hemodynamic performance for patients older than 60 [13]. Mortality rates were recorded to be 3.5% among patients, only 27 out of 51 patients had to undergo reoperation due to SVD, and the rates of reoperation and SVD were considered acceptable. In another study, the Mosaic bioprosthetic valve showed a low incidence of SVD and lasting durability over the course of 13 years [7]. When compared to the Hancock II, the gold standard of porcine valves due to its lower incidence of valve calcification, freedom from SVD was observed to be similar, demonstrating the successful durability of the Mosaic valve [7]. Finally, the bovine BalMedic bioprosthetic valve, designed in China to specifically target valvular heart disease, was found to be suitable for patients undergoing AVR with lowered SVD frequency in younger patients [14]. The freedom from SVD was overall higher in the AVR group at a rate of 90.95%, indicating that the BalMedic valve is an optimal choice [14]. The evidence presented in the aforementioned cases highlights the potential for increased durability in bioprosthetic valves and a decrease in SVD rates during a 14-year follow-up [14]. Our case report adds to this by presenting a unique case of the longest known survival of a patient with a bioprosthetic valve that has not undergone any known structural valve degeneration.

This case report focuses on a 76-year-old patient who had an AVR with an SJM bioprosthetic valve in 1980, indicating that the valve has withstood a duration of more than 42 years without known structural valve degeneration. Upon examination, it was discernible that the patient retained clinical and hemodynamic stability and was free of symptoms associated with valvular dysfunction, allowing for daily activities without restraint. Although a majority of patients younger than 65 experienced deterioration of their bioprosthetic valves within a decade, this patient’s valve has not undergone SVD despite the patient being 33 years old at the time of the AVR [4]. This case report adds to the data on bioprosthetic valves that can withstand SVD for long durations of time. This can also prove beneficial by highlighting aspects of the valve that are more resilient to SVD, which can be used to redesign bioprosthetic valves especially targeting younger patients in an effort to reduce reoperation rates and the need for long-term anticoagulation therapy [1].

## Conclusions

The evidence of cases such as these can establish the potential for improved durability of bioprosthetic valves and expand their use in younger patient populations. The patient that is the focus of this case study is a prime example of bioprosthetic longevity post-AVR and may be the longest survival case known. This adds to the scientific literature by providing data that can identify key characteristics indicative of reducing the occurrence of SVD, resulting in prospective improvements in bioprosthetic valve design. Additionally, this case can guide clinicians to consider alternatives to mechanical valves for younger patients, avoiding long-term anticoagulant therapy and reducing the risk of bleeding. Furthermore, this case study demonstrates that bioprosthetic valves used in AVR may be more durable in younger patients than was previously acknowledged, presenting the possibility of the expansion of valve options.
